# Causes of deaths in neonates and children aged 1–59 months in Nigeria: verbal autopsy findings of 2019 Verbal and Social Autopsy study

**DOI:** 10.1186/s12889-022-13507-z

**Published:** 2022-06-06

**Authors:** Adeyinka Odejimi, John Quinley, George Ikechi Eluwa, Michael Kunnuji, Robinson Daniel Wammanda, William Weiss, Femi James, Mustapha Bello, Adenike Ogunlewe, Rebekah King, Ana Claudia Franca-Koh

**Affiliations:** 1 Department of Health Planning, Research, and Statistics, Federal Ministry of Health, Abuja, Nigeria; 2 Social Solutions International, Rockville, USA; 3 Diadem Consults Ltd, Abuja, Nigeria; 4 Department of Sociology, University of Lagos, Lagos, Nigeria; 5 Department of Paediatrics, Ahmadu Bello University Teaching Hospital, Ahmadu Bello University, Zaria, Nigeria; 6 Department of International Health, Johns Hopkins University, Baltimore, USA; 7 Department of Family Health, Child Health Division, Federal Ministry of Health, Abuja, Nigeria; 8 Department of Paediatrics, University of Maiduguri Teaching Hospital, Maiduguri, Borno State Nigeria; 9 National Population Commission, Abuja, Nigeria

**Keywords:** Verbal autopsy, Neonatal, Under-five mortality, Cause distribution, Child health

## Abstract

**Background:**

Nigeria has one of the highest under-five mortality rates in the world. Identifying the causes of these deaths is crucial to inform changes in policy documents, design and implementation of appropriate interventions to reduce these deaths. This study aimed to provide national and zonal-level estimates of the causes of under-five death in Nigeria in the 2013–2018 periods.

**Methods:**

We conducted retrospective inquiries into the cause of deaths of 948 neonates and 2,127 children aged 1–59 months as identified in the 2018 Nigeria Demographic and Health Survey (NDHS). The verbal autopsy asked about signs and symptoms during the final illness. The Physician Coded Verbal Autopsy (PCVA) and Expert Algorithm Verbal Autopsy (EAVA) methods were employed to assign the immediate and underlying cause of deaths to all cases.

**Result:**

For the analysis, sampling weights were applied to accommodate non-proportional allocation. Boys accounted for 56 percent of neonatal deaths and 51.5 percent of the 1–59-months old deaths. About one-quarter of under-5 mortality was attributed to neonatal deaths, and 50 percent of these neonatal deaths were recorded within 48 h of delivery. Overall, 84 percent of the under-5 deaths were in the northern geopolitical zones. Based on the two methods for case analysis, neonatal infections (sepsis, pneumonia, and meningitis) were responsible for 44 percent of the neonatal deaths, followed by intrapartum injury (PCVA: 21 percent vs. EAVA: 29 percent). The three main causes of death in children aged 1–59 months were malaria (PCVA: 23 percent vs. EAVA: 35 percent), diarrhoea (PCVA: 17 percent vs. EAVA: 23 percent), and pneumonia (PCVA: 10 percent vs. EAVA: 12 percent). In the North West, where the majority of under-5 (1–59 months) deaths were recorded, diarrhoea was the main cause of death (PCVA: 24.3 percent vs. EAVA: 30 percent).

**Conclusion:**

The causes of neonatal and children aged 1–59 months deaths vary across the northern and southern regions. By homing on the specific causes of mortality by region, the study provides crucial information that may be useful in planning appropriately tailored interventions to significantly reduce under-five deaths in Nigeria.

**Supplementary Information:**

The online version contains supplementary material available at 10.1186/s12889-022-13507-z.

## Background

Globally, there has been considerable progress in reducing under-five mortality rate over the last 30 years, with mortality rate declining from 93 per 1000 live births in 1990 to 43 per 1000 live births in 2015. After 2015, the global community transitioned from the Millennium Development Goals to the Sustainable Development Goals (SDGs), with SDG 3.2 targeting an under-five mortality rate reduction to 25 per 1000 live births and neonatal mortality rate to as low as 12 per 1000 live births by 2030 [1].

As of 2019, the global under-five mortality rate had declined to 38 per 1000 live births, with 45 percent of these deaths occurring during the neonatal period. An estimated 53 percent of under-five deaths occur in sub-Saharan Africa [2]. Nigeria accounts for a quarter of all neonatal and child deaths in sub-Saharan Africa and is now the country with the highest number of under-five deaths in the world [2].

The Federal Government of Nigeria has been working continuously to reduce child mortality through the development of policies and strategies, and the implementation of the following interventions and programs: the Midwives Service Schemes [3], the Maternal, Newborn & Child Health Week, Community Based Newborn Care [4]***,*** Saving One Million Lives (SOML) initiative [5], Subsidy Reinvestment and Empowerment Programme for MCH [6], the UN Commission on Life Saving Commodities [7], Essential Newborn Care, Integrated Management of Childhood Illnesses [8], Integrated Community Case Management [8], and the 100 K Helping Babies Survive and Thrive initiative [8]. Based on the 2003 and 2018 Nigeria Demographic and Health Survey (NDHS) findings, Nigeria’s under-five mortality rate declined over the 15 years from 201 per 1000 live births to 132 per 1000 live births. However, the neonatal mortality rate only slightly declined, from 48 per 1000 live births in 2003 to 39 per 1000 live births in 2018 [9, 10].

Nigeria has the world’s highest child mortality rate [2], though wide differences are found across the country’s six geopolitical zones. The North West zone has the highest under-five mortality rate at 187 per 1000 live births, and the South West zone is lowest, at 62 per 1000 live births [10]. The majority of these deaths are due to preventable and treatable conditions [11]. Access to timely and appropriate intervention is crucial to ensuring the reduction of these deaths [11, 12]. The 2018 NDHS report showed that only 39 percent of women delivered in a facility, and just 31 percent of children aged 12–23 months received all their basic vaccinations [10].

Functional civil registration systems with an accurate medical certification of the causes of deaths have been identified as the best source of mortality data, especially for under-five children. However civil registration systems in low- and -middle-income countries, including Nigeria, are weak, and implementation is undermined by lack of funding, staff shortages, and weak infrastructure [13, 14]. Nigerian Civil Registration and Vital Statistics (CRVS) assessment revealed that the system cannot currently produce reliable mortality statistics for monitoring SDG 3.2 [15].

Verbal autopsy for under-5 deaths can provide information that could aid policymaking, planning, and evidence-based action. Verbal autopsy—a retrospective inquiry with the caregivers of the deceased regarding the signs and symptoms of the fatal illness has been identified as a useful tool in resource-limited settings where under-five mortality is unacceptably high [16–18], and its effectiveness has been documented in studies [19, 20]. Findings from verbal autopsies provide additional valuable information which can help policymakers, development partners, and other relevant stakeholders with better understanding of the epidemiological trends of under-five mortality and develop appropriate interventions.

The Verbal and Social Autopsy (VASA) Survey 2019 for under-five deaths was conducted based on deaths identified in the 2018 NDHS. The conduct of the 2019 VASA for the period under survey (2013–2018) aimed to provide national and zonal level estimates of the causes of under-five death in Nigeria and this is pertinent to the availability of up-to-date, comprehensive data on the cause distribution of under-five deaths to guide scarce resource allocation.

## Methods and materials

### Study design and sample

This study is a cross-sectional nationally representative survey of all the 36 states of Nigeria and the Federal Capital Territory based on the 2018 NDHS. The VASA study sampled household ride on the platform of 2018 NDHS multi stage sampling procedure. In the first stage, a total of 1,400 Enumeration Areas (EAs) were selected on a probability proportionate to size basis within urban and rural areas of Nigeria's states (with double the number of EAs in Kano and Lagos due to their large populations).

In the second stage, 30 households were selected from each cluster, resulting in a target sample of 42,000 households.

**Fig. 1:**
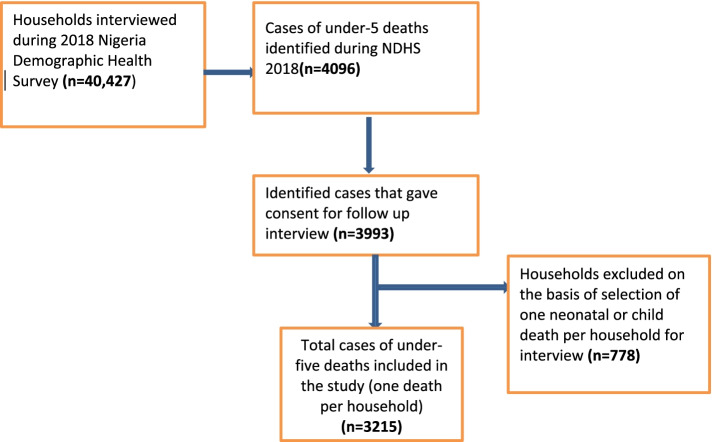
Sampling procedure for selection of households for under-five deaths

As described in Fig. 1, Out of the 40,427 households successfully interviewed during the 2018 NDHS, 4,096 cases of under-five deaths were reported within the period of 2013–2018. Consent for follow-up interviews about the signs and symptoms of the fatal illnesses that led to the deaths was obtained from households covering 3,993 of these deaths in households, where two or more deaths were reported over the sampled period, only the most recent death was selected for interview. This aimed to prevent overlapping of the environmental and health system factors of deaths within the same households and reduce burden of interview for the single family. Hence, 778 households were excluded, and 3215 under five deaths (one per household) were selected for the interview.

### Study instrument

The 2019 VASA survey instrument was based on the 2016 WHO Global Standard Verbal Autopsy Questionnaire [21]. For social autopsy topics not included in the WHO standard, we added questions from the Nigerian 2014 VASA survey instrument, which was based on the Child Health Epidemiology Reference Group social autopsy questionnaire [22]. The final study instrument consisted of 15 modules which were grouped into three main sections. The first section applied to all deaths and had five modules: (1) general information for all deaths, (2) history of injuries/accidents for all deaths, (3) care-seeking during the fatal illness for neonates and child deaths, (4) medical records and mother’s HIV status, and (5) Social Capital. The modules specific to neonatal deaths included the following: (1) validation of neonatal death versus stillbirth status, (2) health history for neonates, (3) signs and symptoms for neonates, (4) pregnancy, labour, and delivery history for neonates, (5) stillbirths, and (6) newborn routine care. The modules specific to deaths among children aged 1–59 months included the following: (1) health history, (2) medical history, (3) signs and symptoms, as well as (4) routine care for children aged 1–59 months.

The study instrument was translated to the three major local languages: Yoruba, Igbo, and Hausa. The translated instruments were back translated to English to confirm the validity and content of the translations. The survey instrument and final translations were programmed into the Census and Survey Processing System (CSPro) software application that enabled the interviewers to capture data directly into a netbook computer.

### Recruitment and training of interviewers

A total of 53 field researchers and 15 supervisors with the following qualifications were recruited for the study: experienced in quantitative data collection, familiar with the study setting and fluent in both English and at least one of the three local languages for the interview (many of the interviewers had also participated in the 2018 NDHS). Also, to ensure the generation of quality data, non-medical experienced field researchers were recruited for data collection to avoid prompting the respondents toward presumptive diagnosis. They underwent 17 days of training on how to conduct the verbal autopsy as well as three days of field practice. The training covered the data collection instrument, interviewing techniques, ethical principles, eligibility criteria, and use of a computer-aided personal interview device to capture the data.

### Data collection and quality assurance

Data were collected from October to December 2019. The trained data collectors visited the selected households to interview the mother/caregiver of the deceased of each selected case. Where the mother was not available or not involved in the care of the deceased during the fatal illness, the person directly involved in caring for the deceased during the child's illness was interviewed. The interviewers obtained informed consent before administering the study instrument.

Data quality was ensured throughout the data collection process through CSPro automated checks on a range of values, including consistency checks of responses on the collected data. Also, the supervisors with the field editor ensure completeness of every interview before final submission.

### Analysis

The 2019 VASA employed two methods for the analysis of survey data to determine the cause of death: Physician-Coded Verbal Autopsy (PCVA) and Expert Algorithm Verbal Autopsy (EAVA). For the PCVA, two in-country physicians were trained on the use of guidelines for coding the cause of death from verbal autopsy interviews based on the International Classification of Diseases (ICD) 10 principle [23] and were provided with minimum diagnostic criteria for ascribing the cause of death. The purpose of the minimum diagnostic criteria was to ensure that the physicians aligned with international standards and eliminate possible bias in diagnosis based on the physician’s knowledge of symptoms and signs ascribed to certain diagnoses in the environment. Each physician independently reviewed all the submitted interviews and assigned the cause of death based on the minimum criteria along with his or her clinical judgment. The physicians also considered the relevant information in the narratives in the open-ended section of the questionnaire in assigning the cause of death. Physicians assigned main, underlying, and contributing causes of deaths for each case and completed the international standard death certificate accordingly. The two physicians had review meetings to compare notes on the assigned causes of deaths and reached a consensus on the final causes of deaths for all the cases. A senior paediatrician reviewed their submissions to ensure compliance with minimum diagnostic criteria guidelines.

For the EAVA, verbal autopsy experts developed a computerized code (“expert algorithm”) following criteria used for previous verbal autopsy studies [18, 19] to automate the distribution of the causes of death from verbal autopsy interview responses. The ascribed direct causes of deaths were based on hierarchical order, meaning that once a case meets the criteria for one diagnosis, diagnoses further down the hierarchy are no longer considered. Causes with the clearest symptoms (e.g., injuries, congenital abnormalities) are placed high in the hierarchy while those that are more ambiguous (e.g., sepsis) are placed lower. The expert algorithm cannot include diagnoses that are not on its list. Any cases that do not meet any of the criteria for a diagnosis in the hierarchy are classified as unspecified.

The data analysis was carried out using the STATA version 14.2 statistical package. For demographic characteristics and causes of deaths, percentages, mean, and medians were used to present the findings. Sampling weights derived from the 2018 NDHS were applied in the analysis to correct for non-proportional allocation of the NDHS sample to the states and zones. The chi-square test statistic was used to test differences in the distribution of categorical variables across diagnostic methods, while the Wilcoxon rank-sum test was used to compare differences in continuous variables across diagnostic methods. To assess the level of agreement between diagnostic methods, the Kappa statistic was conducted for the cause of deaths of both neonates and children aged 1–59 months.

### Ethical approval

Ethical approval was obtained from the National Health Research Ethics Committee of the Federal Ministry of Health in Nigeria and the Social Solutions International Inc. Institutional Review Board of Rockville, Maryland, USA. The researchers sought and obtained informed consents from the participating mothers and care givers during the 2018 NDHS data collection and before interviews were conducted during the 2019 VASA study.

## Results

### Response rate

Out of the 3,215 households sampled, a total of 3,075 interviews were completed for a response rate of 95.6 percent. The majority of respondents were mothers of the deceased, and 99.4 percent of the respondents lived with the deceased during the fatal illness that led to death.

### Demographic characteristic of the survey sample

During the data collection, the respondents were asked the following questions “never breathe, never moved and never cried after delivery “to clearly identify cases of stillbirths from neonatal deaths. About 20 percent (194) of what were reported to be neonatal deaths in the NDHS were found to be stillbirths in this study. The study was not designed to determine the underlying causes of stillbirth, so these cases were excluded from the diagnosis by the two methods. Table 1 illustrates the demographic characteristics of the 2881 neonates and children aged 1–59 months who died. Of the total deaths, 26.2 percent (754) were neonatal deaths and 73.8 percent (2,127) were 1–59 months old deaths.

**Table 1:** Demographic characteristics of the under-5 deaths

Characteristics		Sampled deaths		
		Neonatal deaths	1–59 months deaths	**Total deaths**
		% (n)	% (n)	% (n)
**Age**				
Median age		1 day	19 months	2881
**Sex**				
Female		44.4 (335)	48.5 (1031)	47.4 (1366)
Male		55.6 (419)	51.5 (1096)	52.6 (1515)
**Zones (States)**				
North West	(Jigawa, Kaduna, Kano, Katsina, Kebbi, Sokoto, Zamfara)	39.3 (296)	49.0 (1042)	46.4 (1338)
North East	(Adamawa, Bauchi, Borno, Gombe, Taraba, Yobe)	25.5 (192)	22.5 (479)	23.3 (671)
North Central	(FCT, Benue, Kogi, Kwara, Nasarawa, Niger, Plateau)	15.4 (116)	14.3 (303)	14.5 (419)
South West	(Ekiti, Ondo, Lagos, Osun, Oyo, Ogun)	5.0 (38)	3.1 (65)	3.6 (103)
South East	(Abia, Anambra, Ebonyi, Enugu, Imo)	8.0 (60)	6.5 (138)	6.9 (198)
South South	(Akwa Ibom, Bayelsa, Cross River, Delta, Edo, Rivers)	6.9 (52)	4.7 (100)	5.3 (152)

The average recall period between the interview and death was 39 months (3.25 years). The median age of death for neonates and children aged 1–59 months was 1 day (interquartile range [IQR] 0 – 6)and 19 months (9 – 28), respectively. Males accounted for 55.6 percent of neonatal deaths and 51.5 percent of 1–59 months old deaths.

Nigeria is divided into six geopolitical zones with three in the north (North West, North East, and North Central) and three in the south (South South, South East, and South West). The majority of the under-five deaths occurred in the northern zones. The North West and North East zones together accounted for 65 percent and 72 percent of the deaths in the neonates and children aged 1–59 months deaths surveyed, respectively.

### Cause-specific neonatal mortality

**Fig. 2:**
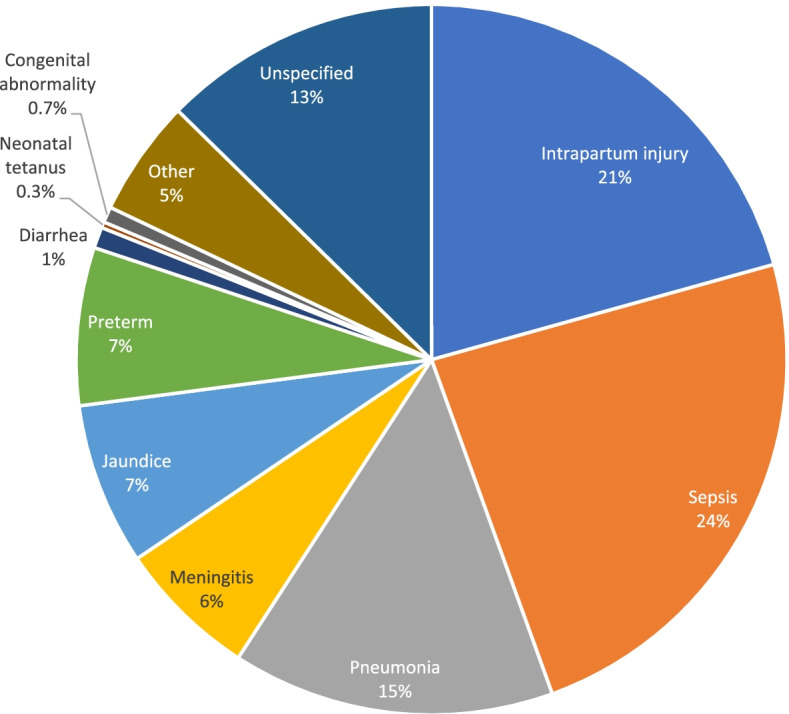
Physician coded verbal autopsy, causes of neonatal deaths (weighted)

**Fig. 3:**
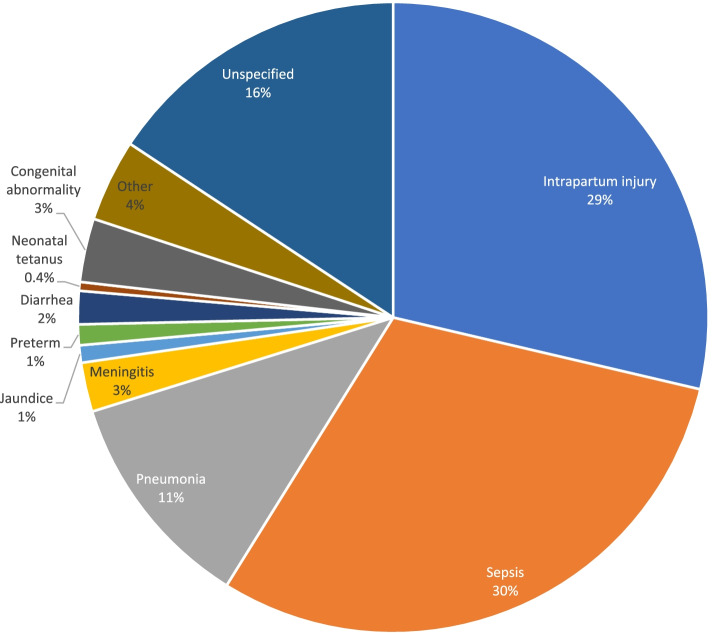
Expert algorithm verbal autopsy, causes of neonatal deaths (weighted)

Figures 2 and 3 highlight the main causes of neonatal mortality using PCVA and EAVA analysis methods. Overall, neonatal infections and intrapartum injury (previously known as Birth Asphyxia) were the predominant causes of neonatal mortality. Neonatal infections (sepsis, pneumonia, and meningitis) accounted for 44 percent of deaths by both PCVA and EAVA, and intrapartum injury accounted for 21 percent and 29 percent by PCVA and EAVA, respectively. Neonatal tetanus, at 0.27percent (PCVA) and 0.45 percent (EAVA), was the least common among specified causes of neonatal death. The Kappa level of agreement for all causes of neonatal death by both methods was 0.55 (95% CI: 0.49 – 0.61).

### Trends in causes of neonatal deaths

Causes of death in this study were compared with a similar verbal autopsy survey conducted in country in 2014 as a follow up to 2013 NDHS.

**Fig. 4:**
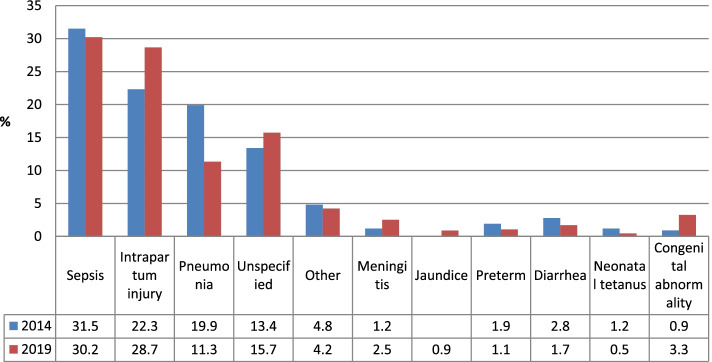
Causes of deaths in neonates

Figure 4 below depicts the trends in the causes of deaths from 2014 to 2019. In 2014, the three leading causes of neonatal deaths using PCVA were sepsis, Intrapartum injury and pneumonia. In the same vein, the leading causes of death in 2019 were sepsis, Intrapartum injury and unspecified. Irrespective of the pattern of occurrence, the leading causes of deaths in 2014 and 2019 were similar.

### Age disparity in causes of neonatal deaths and children aged 1–59 months old

Table 2 shows the causes of death during the neonatal period and1-59 months old causes of death. Intrapartum injury was the leading cause of death within 24 h of birth for both PCVA (41 percent) and EAVA (50 percent). Beyond the first day of birth, severe infection was the leading cause of death. Neonatal deaths caused by “Other” include cases suggestive of haemorrhagic disease of the newborn, skin infection, and sudden death. In children aged 1-59 months, the leading cause of deaths for aged 1-11 months was diarrhoea for PCVA (21.5 percent) and malaria for EAVA (31.7percent). However, for recorded deaths for children aged 12–59 months, Malaria was the leading cause of death as assigned by PCVA (23.8 percent) and EAVA (37.0 percent).

**Table 2:** Causes of neonatal deaths by age (weighted)

	Physician Coded Verbal Autopsy					Expert Algorithm Verbal Autopsy				
	Causes of deaths (%)					Causes of deaths (%)				
**Age Group**	**Intrapartum injury (95% CI)**	**Preterm (95% CI)**	**Severe infection (95% CI)**	**Other (95% CI)**	***P*-value**	**Intrapartum injury (95% CI)**	**Preterm (95% CI)**	**Severe infection (95% CI)**	**Others (95% CI)**	***P*-value**
0 days	40.5 (34.7 – 46.9)	11.3 (7.9 – 15.8)	18.8 (14.4 – 24.1)	29.3 (24.0 – 35.3)		50.4 (44.1 – 56.5)	0.8 (0.3 – 3.3)	28.2 (22.8 – 34.0)	20.6 (16.1- 26.2)	
1 day	26.1 (19.1 – 35.1)	8.7 (4.7 – 15.4)	48.7 (39.2 – 57.4)	16.5(11.0 – 24.8)		34.5 (26.2 – 43.4)	0.5 (0.0 – 6.3)	40.5 (32.3 – 50.1)	24.1 (17.3 – 33.0)	
2–6 days	7.7 (4.8 – 12.2)	3.8 (2.0 – 7.7)	62.5 (55.6 – 68.8)	26.0 (20.3 – 32.3)		18.3 (13.6 – 24.1)	1.4 (0.5 – 4.5)	52.9 (46.1 –59.7)	27.4 (21.6 – 33.7)	
7–27 days	3.9 (1.8 – 8.0)	4.0 (2.0 – 8.4)	59.0 (51.5 – 66.1)	32.9 (26.4 – 40.4)	< 0.0001	6.3 (3.5 – 11.0)	1.2 (0.2 – 4.4)	58.4 (51.0 – 65.6)	34.1 (27.5 – 41.6)	< 0.0001
**1–59 months**	**Diarrhoea (95% CI)**	**Pneumonia (95% CI)**	**Malaria (95% CI)**	**Other (95% CI)**		**Diarrheal (95% CI)**	**Pneumonia (95% CI)**	**Malaria (95% CI)**	**Other (95% CI)**	
1–11 months	21.5 (18.4 – 24.9)	12.6 (10.2 – 15.5)	20.2 (17.3 – 23.6)	45.6 (41.8 – 49.6)		23.5 (20.3 – 27.0)	15.1 (12.5 – 18.1)	31.7 (28.1 – 35.4)	29.8 (26.3 – 33.5)	
1–5 years	21.4 (19.4 – 23.5)	8.7 (7.4 – 10.2)	23.8 (21.7 – 26.0)	46.2 (43.7 – 48.7)	0.027	28.8 (26.6 – 31.2)	11.1 (9.6 – 12.8)	37.0 (34.6 – 39.4)	23.1 (21.1 – 25.3)	0.002

*CI* Confidence Interval; “Other” for neonatal deaths: include cases suggestive of haemorrhagic disease, skin infections and sudden deaths, and jaundice. “Other” for age 1–59 months include: severe anaemia (without a specific cause), sickle cell anaemia, haemorrhagic fever, skin infections and sudden death

### Cause-specific mortality for children aged 1–59 months

**Fig. 5:**
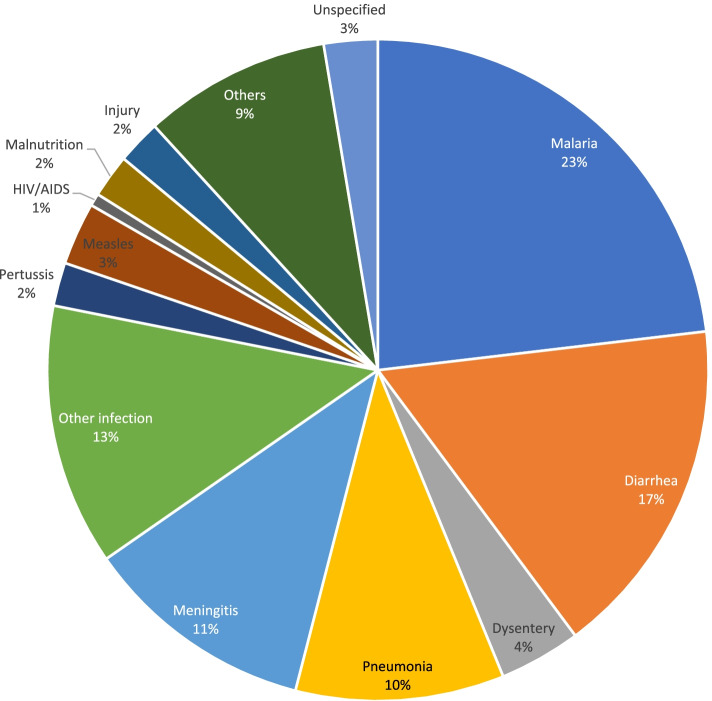
Physician coded verbal autopsy, causes of child 1–59 months deaths (weighted)

**Fig. 6:**
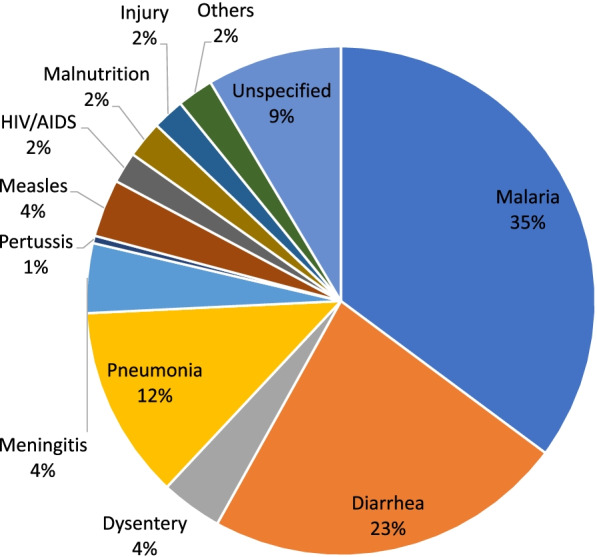
Expert algorithm verbal autopsy, causes of child 1–59 months deaths (weighted)

Figures 5 and 6 illustrate the main causes of deaths in children aged 1–59 months as assigned by PCVA and EAVA. Malaria (23 percent and 35 percent) and diarrhoea (17 percent and 23 percent) were ranked as the two leading causes of death for PCVA and EAVA, respectively. Other infections (13 percent) and pneumonia (12 percent) ranked as the third leading cause of death as assigned by PCVA and EAVA, respectively. For vaccine-preventable diseases, measles (3 percent and 4 percent) and pertussis (2 percent and 1 percent) contributed less than 5 percent of deaths as determined by PCVA and EAVA, respectively. Malnutrition, which might be an underlying or contributory factor to childhood illnesses, was found to be the direct cause of a small percentage of deaths of children 1–59 months (2 percent for both PCVA and EAVA). Causes of deaths categorized as “others” for children aged 1-59 month old in these figures are severe anaemia (without a specific cause), sickle cell anaemia, haemorrhagic fever, and unexplained sudden death. The Kappa level of agreement for all causes of 1–59 months old deaths (including “others”) by both methods was 0.50 (95% CI:0.46 – 0.54).

### Trends in the causes of child 1–59 months old deaths

**Fig. 7:**
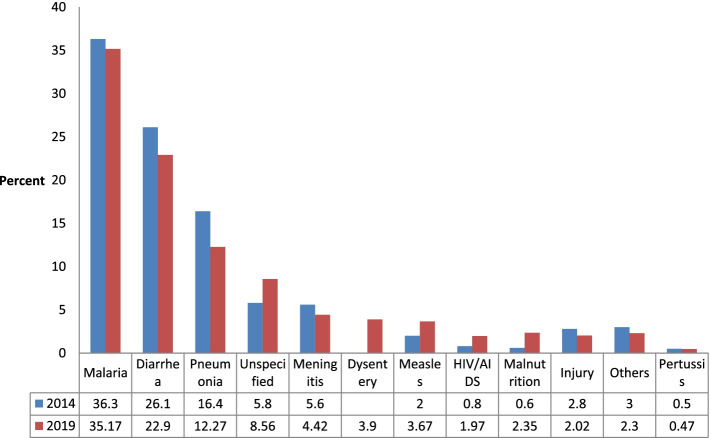
Causes of death in children 1–59 months old

The pattern of the causes of deaths for children 1–59 months old in the last 5 years were similar (Fig. 7). The VASA 2014 & 2019 demonstrate that the three leading causes of these deaths were malaria, diarrhoea, and pneumonia.

### Zonal-level cause estimates of the neonatal deaths

Table 3 shows the cause distribution of neonatal deaths across the six geopolitical zones in the country as found by the two methods. In northern Nigeria, severe neonatal infection was the leading cause of death, followed by intrapartum injury, as determined by PCVA and EAVA. This is in contrast to two of the three southern zones, where the intrapartum injury was the leading cause. In South East and South West, the intrapartum injury was the most common cause of death by both methods (PCVA: 32.4 percent and EAVA: 37.4 percent for South East; and PCVA: 39.3 percent and EAVA: 39.6 percent for South West). However, in South South, the most common cause of death was severe infection (PCVA: 41.7 percent and EAVA: 43.6 percent).

**Table 3:** Causes of neonatal deaths by zone (weighted)

	Physician Coded Verbal Autopsy Causes of deaths (%)				Expert Algorithm Verbal Autopsy Causes of deaths (%)			
Zone	Intrapartum injury (95% CI)	Preterm (95% CI)	Severe infection (95% CI)	Other (95% CI)	Intrapartum injury (95% CI)	Preterm (95% CI)	Severe infection (95% CI)	Others (95% CI)
North Central	16.7 (10.1 – 26.3)	6.7 (2.9 – 14.3)	45.0 (34.7 – 55.8)	31.7 (22.6 – 42.4)	32.6 (23.4 – 43.3)	0.4 (0.0 – 0.1)	38.3 (28.5 – 49.1)	28.7 (20.0 – 39.3)
North East	18.3 (13.1 – 25.0)	4.0 (1.9 – 8.4)	53.7 (46.0 – 61.2)	24.0 (18.0 – 31.2)	21.8 16.1 – 28.8)	1.3 (0.0 – 0.1)	51.2 (43.5 – 58.8)	25.7 (19.6 – 33.0)
North West	18.9 (15.2 – 23.3)	7.3 (5.1 – 10.5)	47.1 (42.0 – 52.3)	26.7 (22.4.- 31.5)	29.0 (24.6 – 33.9)	1.22 (0.3 – 4.9)	46.2 (41.1 – 51.4)	23.6 (19.5 – 28.2)
South East	32.4 (20.9 – 46.4)	3.2 (0.7 – 13.8)	21.7 (11.8 – 34.4)	43.5 (30.5 – 57.4)	37.4 (25.2 – 51.5)	0	30.5 (19.3 – 44.5)	32.1 (20.7 – 46.2)
South South	22.0 (12.0 – 36.7)	8.3 (3.0 – 21.2)	41.7 (28.0 – 56.7)	28.1 (16.7 – 43.2)	23.8 (13.4 – 38.7)	0.9 (0.0 – 1.7)	43.6 (29.7 – 58.6)	31.8 (19.6 – 47.0)
South West	39.3 (25.2 – 55.5)	24.5 (13.4 – 40.6)	21.3 (11.0 – 37.1)	14.9 (6.6 – 30.0)	39.6 (25.4 – 55.7)	1.5 (0.1 – 17.3)	25.0 (13.7 – 41.0)	33.9 (20.7 – 50.2)
Total	20.7 (17.9 – 23.8)	7.2 (5.5 – 9.3)	44.9 (41.3 – 48.5)	27.3 (24.2 – 30.6)	28.7 (25.5 – 32.0)	1.1 (0.5 – 2.1)	44.1 (40.5 – 47.7)	26.2 (23.2 – 29.5)

*CI* Confidence interval; “Other”: cases suggestive of haemorrhagic disease of the newborn, skin infection, sudden death, and jaundice

Table 4 shows the zonal distribution of causes of death of children aged 1–59 months. Across the six geopolitical zones, malaria was the leading cause of death for both PCVA and EAVA. However, in the North West, which has the highest under-five mortality, the PCVA, and EAVA findings diverged, with the former finding diarrhoea to be the leading cause of death (24.3 percent) and the latter finding malaria to be the leading cause (34.1 percent). diarrhoea was the second leading cause of death across all zones by both PCVA and EAVA except in South West, where pneumonia was the second leading cause of death by PCVA (13.9 percent).

**Table 4:** Zonal distribution of causes of 1–59 months children deaths (weighted)

	Physician Coded Verbal Autopsy Causes of deaths (%)				*P*-value	Expert Algorithm Verbal Autopsy Causes of deaths (%)				*P*-value
Zone	Diarrhoea (95% CI)	Pneumonia (95% CI)	Malaria (95% CI)	Other (95% CI)		Diarrhoea (95% CI)	Pneumonia (95% CI)	Malaria (95% CI)	Others (95% CI)	
North Central	15.3 (11.1 – 20.7)	12.2 (8.4 – 17.1)	24.4 (19.3 – 30.6)	48.0 (41.5 – 54.7)		19.1 (14.2 – 24.6)	20.1 (15.3 – 25.9)	36.1 (30.0 – 42.7)	25.0 (19.7- 31.1)	
North East	21.7 (17.9 – 26.0)	9.1 (6.7 – 12.4)	27.2 (23.0 – 31.7)	41.8 (37.2- 46.9)		29.0 (24.7 – 33.5)	11.1 (8.3 – 14.5)	36.1 (31.7 – 41.1)	23.8 (19.9 – 28.2)	
North West	24.3 (22.0 – 27.0)	9.3 (7.8 – 11.0)	22.0 (19.8 – 24.5)	44.3 (41.6 – 47.1)		30.0 (27.5 – 32.6)	10.9 (9.3 – 12.8)	34.1 (31.5 – 36.8)	25.0 (22.6 – 27.5)	
South East	12.4 (7.7 – 20.2)	12.4 (7.4 – 19.7)	13.3 (8.1 – 20.8)	61.9 (52.5 – 70.3)		25.9 (18.6 – 34.7)	16.2 (10.5 – 24.2)	36.3 (27.9 – 45.4)	21.8 (15.1 – 30.3)	
South South	15 .0 (9.4 – 23.7)	8 .0 (3.9 – 15.0)	18.0 (11.6 – 26.7)	59.0 (49.1 – 68.3)		15.4 (9.5 – 23.9)	14.2 (8.6 – 22.6)	35.4 (26.7 – 45.3)	35.0 (26.3 – 44.8)	
South West	11.4 (6.2 – 20.7)	13.9 (8.0 – 23.5)	26.6 (18.0 – 37.3)	48.1 (37.1 – 58.7)	< 0.00001	17.7 (10.9 – 27.8)	9.0 (4.4 – 17.6)	48.7 (37.9 – 59.6)	25.3 (16.3 – 35.1)	< 0.0001

*CI* Confidence Interval; “Other” include severe anaemia (without a specific cause), sickle cell anaemia, haemorrhagic fever, and unexplained sudden death

## Discussion

The study provides information on the causes of neonatal deaths across different age groups during the neonatal period as well as the distribution of causes of death in children aged 1–59 months old.

The Federal Government of Nigeria has made several efforts to strengthen death certification and registration in the country. As part of the effort, automation and digitalization of CRVS was launched to capture community-level births and mortality. In the same vein, the Maternal and Perinatal Death Surveillance and Response initiative, launched under the purview of the Federal Ministry of Health, aimed to systematically audit facility-based maternal and perinatal mortality. Despite such efforts, many birth and deaths go unregistered especially in rural communities. Only 32 percent of the deaths that occur in rural areas were registered compared to 60 percent of registered birth in urban communities (NDHS 2018). The poor implementation of the International Classification of Diseases across facilities also poses a challenge to standardizing medical certification of the causes of death. Consequently, comprehensive information on the causes of death for all age groups, including under-5, is elusive. Identifying the causes and determinants of mortality is critical to ensure appropriate design and implementation of interventions to improve the health status of the populace.

Surprisingly, about 20% of the cases identified to be neonatal deaths during NDHS 2018 were found to be still births during Verbal autopsy. There were similar findings in previous verbal autopsy for under-five deaths that selected the study sample from NDHS [18, 19]. This might be due to verbal autopsy method of eliciting for information during interview to adequately differentiate still births from neonatal deaths.

Neonatal and 1–59-months-old deaths are more common in boys than girls. Male accounted for about 55.6 percent of neonatal deaths and 51.5 percent of deaths in children aged 1–59 months. Other studies have documented similar findings, citing biological difference as the possible factor for this gender variation for under-five deaths [24, 25].

This study found that neonatal deaths contributed to 26.2 percent of under-five mortality, the majority being in the northern zones. Also, about 50 percent of these neonatal deaths occur in the first two days after delivery. The finding aligns with WHO reports [26] that many neonatal deaths occur in the first week of life, and that the first two days after birth are the most critical for a child’s survival.Poor access to skilled attendance during labour and immediately after delivery, absence of antenatal care, lack of post natal care in many instances especially in the northern part of Nigeria where home delivery is the norm with poor cord care practices might have contributed to the high neonatal deaths within 2 days of delivery.

About 40% of all neonatal deaths in this study were found in the North West compared to 20% in the southern zones. High neonatal mortality in the North West maybe linked to poor antenatal care attendance with only 18% of births assisted by skilled birth attendants and about 18% of newborn receiving postnatal care within 48 h of delivery. In contrast, in the southern zones 85% of the births were assisted by skilled birth attendants and 72% of the newborn had postnatal care within 48 h of delivery [10].

Despite the low cost proven interventions, neonatal infections were responsible for about 44% of the deaths that occurred within 27 days of life. In contrast to the study finding, neonatal infection accounted for 17.6% of neonatal deaths in Low- income and -Middle Income countries [27]. The high neonatal infection in this study might be linked to poor health seeking behaviour among pregnant women in the northern region where many of the neonatal deaths occurred. Majority of the women rarely visit health facilities for antenatal care for comprehensive maternal–fetal care, timely detection of maternal infections and manage appropriately. Likewise, there is missed opportunity during peripartum where majority of pregnant women delivered at home and rarely visit health facilities for postnatal care. Poor utilization of health facilities during antenatal and peripartum period will result in lack of access to specialised care for the newborn as required.

The low female literacy level may serves as contributing factors to high neonatal infection. Hence, uneducated mothers may not be able to easily identify danger signs and infections related symptoms in the newborn that will necessitate seeking immediate medical attention. Where symptoms are identified, many resolve to local treatment.

Preterm delivery and or low birth weight have been implicated among other factors responsible for neonatal infections [28]. However, there is possibility of underreporting of the preterm delivery especially in the study environment where many could not ascertain the date of menstrual period and birth weight of the newborn for delivery that take place outside the health facility could not be ascertain.

II Intrapartum injury caused half of the neonatal deaths in the first 2 days of life and was identified as the second leading cause of death in the first 27 days of life. Studies conducted in other resource-limited settings revealed similar findings. For example, in a study conducted in the Indian state of Bihar (2019), the intrapartum injury was the most common cause of death for neonates that died within 24 h of life [29]. The Nigeria 2019 VASA study found that prematurity caused 7 percent and 1 percent of neonatal deaths as assigned by PCVA and EAVA, respectively. The expert algorithm considered prematurity lower in the hierarchical order while PCVA noted the cause of deaths in preterm babies was mainly due to associated complications. The latter finding might account for the higher cause distribution assigned to prematurity by PCVA. Similar studies conducted in Egypt and Pakistan reported intrapartum injury, neonatal infections, and prematurity as the three leading causes of neonatal deaths, regardless of the age of the neonates [30, 31]. Likewise, the global estimate placed preterm birth as the leading cause of death worldwide. The low proportion of preterm as the cause of deaths in neonate in this study might be due to underreporting as few women report on pregnancy of 8 months or less. Secondly, this study report country specific primary data and be better understood in context- a country where health seeking behaviour is generally low and an average facility delivery of 39% and many are likely to seek facility delivery if there is pregnancy complications as documented by Singh et al. in 2014 [32].

The uptake of two doses of the tetanus-diphtheria vaccine during pregnancy has been identified as a cost-effective intervention for the survival of the newborn [33]. It is noteworthy that vaccine-preventable disease like tetanus was one of the lowest causes of neonatal deaths in this study. This reflects possible improvement in the uptake of the tetanus-diphtheria immunization during pregnancy and the effectiveness of the national immunization program. In Nigeria, the proportion of tetanus toxoid uptake by pregnant women increased from 40 percent in 2003 to 62 percent in 2018 [10]. The study reveals neonatal deaths due to tetanus infection to be relatively small. This does not necessarily connote low tetanus infections among the surviving newborns.

For those deceased aged 1–59 months, both diagnosis methods found the main causes of deaths to be malaria, diarrhoea, and pneumonia, in that order. It is also noted by PCVA method that the leading cause of death during infancy (age 1-11 months) was diarrhoea. Many of the children especially from age 6 month undergo weaning. Hence they are prone to faeco-oral related illnesses including diarhhoea. The government has prioritized prevention and prompt management of malaria cases for vulnerable groups, including under-five children, by providing free long-lasting insecticidal nets during antenatal visits for pregnant women and during immunization visits for children under 1-year-old. The current findings did not show an appreciable decline in malaria as the leading cause of death compared to the 2014 study by EAVA (35 percent) [18]. However, the PCVA showed that only 23 percent of child deaths were due to malaria. The disparity between the two methods may be due to variation in the criteria for assigning malaria as the cause of death. The PCVA considered the presence of fever with a positive malaria test to assign malaria as the cause of death. However, information on diagnostic tests is not included in the EAVA, and this was not asked in the 2014 study. The discrepancy notwithstanding, malaria is a major contributor to under-five deaths, and the country needs to consider free malaria treatment for children as part of a package of key interventions to improve survival rates.

The Essential Newborn Care Course is designed to build the capacity of frontline health workers to care for the major causes of newborns deaths [34]. In line with WHO’s recommendation, this has been recently expanded to accommodate a simplified regimen for managing possible serious bacterial infections, but implementation is at varying levels in different states and will need to be scaled up to cover more health workers. Integrated Community Case Management (ICCM) is an initiative to enable treatment and referral of the main under-five killer diseases—pneumonia, diarrhoea, and malaria—at the community, level using less-skilled workers. Given this study’s findings that malaria is foremost among the causes of deaths among children aged 1–59 months (in all regions except for North West, where diarrhoea is the primary cause), there may be a need to review the ICCM interventions to include free malaria treatment and administration of seasonal chemoprevention to under-five children, especially in areas with high burden of malaria.

Nigeria has adopted an Integrated Management of Childhood Illnesses (IMCI) approach to strengthen the capacity of health workers in the facility to assess, classify, and treat under-five children using simple algorithms. IMCI also covers the identification of danger signs and referral, including appropriate follow-up. If a referral is deemed necessary for children assessed using the ICCM algorithm, they will be sent to a facility for care by a skilled health worker who will use the IMCI algorithm; as such, IMCI should also be aggressively scaled up.

There are variations across the geopolitical zones in the country on the predominant causes of under-five deaths and understanding these differences will enhance the effective design of appropriate interventions for each zone. For example, malaria is a leading cause of death in children aged 1–59 months in Nigeria. However, diarrhoea was accountable for one-quarter of the deaths of children aged 1–59 months in the North West.

Poor hygiene and sanitation, including poor access to potable water are the typical causes of diarrhoea. Hence, interventions to improve sanitation, as well as ensure availability and access to potable water should be implemented especially for the North West zone.

### Limitations of the study

The main limitation of the study was the recall period of five years for signs and symptoms that may have led to a child’s death. However, experienced data collectors who were skilled in eliciting information were deployed to mitigate this. Even given an accurate history of symptoms, different methods of verbal autopsy analysis give somewhat different results, as shown by the variation in expert algorithm and physician coding methods in this study. In the absence of an actual autopsy or medical diagnosis there is no gold standard to clearly indicate which method is superior overall. This lack of precision is mitigated by a focus in the analysis on broad patterns of mortality, which are still relevant to public health planning.

## Conclusion

The differences in the prevalent causes of death for children under-5 across Nigeria’s geopolitical zones suggest that programs must be tailored to address these causes. It also allows the prioritization and distribution of scarce health resources and helps guide the development of health programs within each zone. Deaths attributed to vaccine-preventable diseases were low, and this may be attributed to the sustained effort of the government on the national program on immunization.

The future direction for tracking cause of death in these age groups will be directed towards strengthening the vital registration system in Nigeria. If the vital registration system in Nigeria is well established, there will be no need to conduct verbal autopsy after every NDHS. Hence, the future of verbal autopsy in Nigeria should be complementary.

In addition, the country has gathered some experience in clinic based Maternal and Perinatal Death Surveillance and Response (MPDSR) which is a formalized audit system that mandates that every death of mothers and perinates be reported, audited, reviewed and responded to, for subsequent improvement in quality of care. This was piloted in substantial number of Primary Health Care Facilities and in the process of scaling up to higher levels.

Learning from this informed the formulation of a Bill for an act that will make notification of such deaths compulsory with penalties for defaulters while still upholding the “No name, no blame” principle. If the bill is signed into law, the recent inclusion of community level verbal autopsies into MPDSR will guarantee an institutionalization of verbal autopsies till the vital registration system fully picks up in the country.

## Supplementary Information

**Additional file 1.** Expert Algorithm used in the Nigeria 2019 VASA study.

**Additional file 2.** Physician minimum diagnostic criteria used in the Nigeria 2019 VASA study.

## Data Availability

The 2019 VASA questionnaire de-identified datasets generated and analysed during the current study are available from the Nigerian National Population Commission at https://nationalpopulation.gov.ng/category/publications/. Further information on cause of death analysis methods and results from this study are available from the corresponding author on reasonable request.

## Abbreviations

CRVS: Civil Registration and Vital Statistics
CSpro: Census and Survey Processing System
EAVA: Expert Algorithm Verbal Autopsy
ICCM: Integrated Community Case Management
ICD: International Classification of Diseases
IMCI: Integrated Management of Childhood Illnesses
MCH: Maternal and child health
NDHS: Nigeria demographic household survey
PCVA: Physician-Coded Verbal Autopsy
SBA: Skilled birth attendant
SDG: Sustainable Development Goal
SOML: Saving One Million Lives
VASA: Verbal and Social Autopsy
