# Supplementary material for: Causes of deaths in neonates and children aged 1–59 months in Nigeria: verbal autopsy findings of 2019 Verbal and Social Autopsy study
Source: BMC Public Health. 2022 Jun 6;22:1130. doi: 10.1186/s12889-022-13507-z (PMC9172014; doi:10.1186/s12889-022-13507-z)
Supplement: Supplementary file 1 — Additional file 1. Expert Algorithm used in the Nigeria 2019 VASA study. [file 12889_2022_13507_MOESM1_ESM.docx]

Supplemental material: Expert Algorithm used in the Nigeria 2019 VASA study

**Source**: National Population Commission (NPC) [Nigeria] and CIRCLE, Social Solutions International, Inc. 2020. *Nigeria 2019 Verbal and Social Autopsy Study: Main Report*. Abuja, Nigeria, and Rockville, Maryland, USA: NPC and Social Solutions International, Inc. Annex 3:  Expert algorithm diagnostic criteria p. 139-144

**Annex 3:  Expert algorithm diagnostic criteria**

***Source*:** Kalter HD, Roubanatou A, Koffi A, Black RE. Direct estimates of national neonatal and child cause–specific mortality proportions in Niger by expert algorithm and physician–coded analysis of verbal autopsy interviews. J Glob Health. 2015;5:010415. Online supplementary Document – Verbal autopsy expert algorithms for neonatal and child causes of death and for maternal infection

Neonatal causes of death

Neonatal tetanus

(Age 3–27 days at death AND convulsions or spasms)

AND EITHER

((Able to suckle normally during the first day of life and stopped being able to suckle)

OR (cried within 5 minutes after birth and stopped being able to cry))

Congenital malformation

Gross malformation present at birth

Birth asphyxia

Neonatal respiratory depression: (Did not cry within 5 minutes after birth OR did not breathe immediately after birth)

AND

Neonatal encephalopathy: (Not able to suckle normally in the first day of life OR convulsions/spasms OR lethargy) OR 0 days old at death

Birth injury

Bruises or signs of injury on the body at birth

Preterm delivery with respiratory distress syndrome

(combined with preterm for final cause distribution)

Pregnancy duration less than 9 months

AND (Fast breathing starting on day 0 AND no fever AND no cold to touch)

Meningitis

Fever

AND (bulging fontanelle OR convulsions)

AND (lethargic OR unresponsive/unconscious)

Diarrhoea

More frequent loose or liquid stools than usual

AND more than 4 stools on the day the diarrhoea was most frequent

Pneumonia

(Fast breathing lasting 1 day or more OR difficult breathing lasting 1 day or more and lasting until death)

AND 2 or more of the following 3 signs: (chest indrawing, grunting, never cried OR stopped crying)

Possible diarrhoea (combined with diarrhoea for final cause distribution)

More frequent loose

Possible pneumonia (combined with pneumonia for final cause distribution

Difficult breathing AND VA sepsis AND No VA pneumonia

Sepsis

Fever OR cold to touch

OR 2 or more of the following 7 signs: (fever OR cold to touch, did not suckle normally on the first day of life OR stopped suckling, convulsions, vomited everything, stopped crying, lethargic OR unconscious, chest indrawing OR grunting)

(Note: ‘vomited everything’ was not specified in 2019 and so ‘vomited’ was used.)

Neonatal jaundice

Yellow skin or yellow eyes

AND (stopped being able to suckle normally OR lethargic OR unresponsive/unconscious) AND No fever or hypothermia

Neonatal haemorrhagic syndrome

Bleeding from anywhere AND No fever or cold to touch

Sudden unexplained death

Died suddenly without appearing ill AND No illness signs or symptoms

Preterm delivery

Pregnancy duration less than 8 months

Unspecified (all others)

All VA diagnoses are negative

Child causes of death

Injury

Suffered from motor vehicle accident, fall, drowning, poisoning, venomous bite or sting, burn, violence or other injury

AND (Died 1 day or less after the injury AND the illness lasted 1 day or less) OR (Injury and No other VA diagnosis (except malnutrition allowed)) OR (Injury that was the first illness sign/symptom AND had VA other infection or fever))

AIDS

(Swelling in the armpits OR a whitish rash inside the mouth/on the tongue)

AND 3 or more of the following 6 signs: (limbs became very thin, protruding belly, more frequent loose/liquid stools than usual for more than 30 days, fever or a skin rash for more than 30 days, fast breathing, chest indrawing)

Malnutrition (underlying)

Limbs became very thin during the fatal illness OR had swollen legs or feet during the illness

AND One of these was the first symptom of the illness

Measles

Child's age greater than or equal to 120 days AND rash for 3 or more days

AND fever for 3 or more days AND the rash started on the face

(Note: 2019 VASA did not specify where rash started so rash on ‘face’ or ‘everywhere’ was accepted)

Meningitis

Fever AND (stiff neck OR bulging fontanelle)

Dysentery

More frequent loose or liquid stools than usual AND more than 4 stools on the day with the most stools AND blood in the stools

OR More frequent loose or liquid stools than usual for more than 14 days AND blood in the stools

Diarrhoea

More frequent loose or liquid stools than usual AND more than 4 stools on the day with the most stools AND No blood in the stools

OR More frequent loose or liquid stools than usual for more than 14 days AND No blood in stools

Pertussis

Cough more than 14 days AND (severe cough OR vomited after coughing OR stridor)

(Note: 2019 VASA did not specify that vomiting occurred after coughing so any vomiting was accepted)

Pneumonia

(Cough more than 2 days OR difficult breathing more than 2 days)

AND (Fast breathing more than 2 days OR chest indrawing OR grunting)

Malaria

Fever that continued till death AND was on and off in character

AND No stiff neck AND No bulging fontanelle AND (pallor OR difficult breathing OR convulsions OR unconscious till death)

OR Fever that continued till death AND was severe fever AND No stiff neck AND No bulging fontanelle AND (pallor OR convulsions OR unconscious till death)

Possible dysentery (combined with dysentery for final cause distribution)

More frequent loose or liquid stools than usual AND (fever OR convulsions OR unconscious up till death) AND blood in the stools AND No VA dysentery

Possible diarrhoea (combined with diarrhoea for final cause distribution)

More frequent loose or liquid stools than usual AND (fever OR convulsions OR unconscious up till death) AND No blood in the stools AND No VA diarrhoea

Possible pneumonia or ARI (combined with pneumonia for final cause distribution)

(Cough or difficult breathing) OR (Fast breathing AND (chest indrawing OR stridor OR grunting OR wheezing))

AND (Severe cough OR post–tussive vomiting OR fast breathing OR chest indrawing OR grunting OR stridor OR wheezing OR fever OR convulsions OR unconscious up till death) AND No VA Pertussis AND No VA pneumonia

Haemorrhagic fever

Fever AND (bled from anywhere OR had areas of the skin that turned black)

Other infection

Fever AND (rash on trunk, abdomen or everywhere OR convulsions OR unconscious up till death)

Possible malaria (combined with malaria for final cause distribution)

Fever AND No other VA infectious causes of death

Malnutrition (combined with underlying malnutrition for final cause distribution)

Limbs became very thin during the fatal illness OR had swollen legs or feet during the illness

Unspecified (all others): All VA diagnoses are negative

Verbal autopsy hierarchies: If a death meets the criteria for a diagnosis, then other diagnoses lower in the hierarchy are not considered.

Neonatal cause of death hierarchy

Injury (added for 2019 VASA using same definition as for child injury)

Neonatal tetanus

Congenital malformation

Birth asphyxia or Birth injury

Preterm delivery with respiratory distress syndrome (combined with preterm delivery for final cause distribution)

Meningitis

Diarrhoea

Pneumonia

Possible diarrhoea (combined with diarrhoea for final cause distribution)

Possible pneumonia (combined with pneumonia for final cause distribution)

Sepsis

Neonatal jaundice

Neonatal hemorrhagic syndrome

Sudden unexplained death

Preterm delivery

Unspecified (all others)

Child cause of death hierarchy

Injury

AIDS

Malnutrition (underlying)

Measles

Meningitis

Dysentery

Diarrhoea

Pertussis

Pneumonia

Malaria

Possible dysentery (combined with dysentery for final cause distribution)

Possible diarrhoea (combined with diarrhoea for final cause distribution)

Possible pneumonia (combined with pneumonia for final cause distribution)

Hemorrhagic fever

Other infections

Possible malaria (combined with malaria for final cause distribution)

Malnutrition (combined with underlying malnutrition for final cause distribution)

Unspecified (all others)
