# Supplementary material for: Causes of deaths in neonates and children aged 1–59 months in Nigeria: verbal autopsy findings of 2019 Verbal and Social Autopsy study
Source: BMC Public Health. 2022 Jun 6;22:1130. doi: 10.1186/s12889-022-13507-z (PMC9172014; doi:10.1186/s12889-022-13507-z)
Supplement: Supplementary file 2 — Additional file 2. Physician minimum diagnostic criteria used in the Nigeria 2019 VASA study. [file 12889_2022_13507_MOESM2_ESM.docx]

Supplemental material: Physician minimum diagnostic criteria used in the Nigeria 2019 VASA study

**Source**: National Population Commission (NPC) [Nigeria] and CIRCLE, Social Solutions International, Inc. 2020. *Nigeria 2019 Verbal and Social Autopsy Study: Main Report*. Abuja, Nigeria, and Rockville, Maryland, USA: NPC and Social Solutions International, Inc. Annex 2:  Physician minimum diagnostic criteria p. 136-138

Annex 2:  Physician minimum diagnostic criteria

***Source*:** NPC, FMoH, IIP, USAID. Annex 5: Physician Minimal Diagnostic Criteria. A Verbal/Social Autopsy Study to improve estimates of the Causes and Determinants of Neonatal and Child Mortality, in Nigeria-Final Report (Version March 2016); Pages 131-135.

Neonatal deaths (0-27 days old)

Birth asphyxia

Did not breath immediately after birth OR Did not cry immediately after birth

Birth trauma

Bruises or signs of injury at birth

Congenital malformation

Physical abnormality at the time of delivery

Diarrhoea

More frequent loose or liquid stools than usual

Meningitis (part of ‘Serious Infection’)

Bulging fontanelle OR Spasms or convulsions

Neonatal tetanus

Stopped suckling normally more than 2 days after birth AND Spasms or convulsions

Pneumonia (part of ‘Serious Infection’)

Difficult breathing OR Fast breathing

Preterm delivery

Pregnancy duration less than 8 months OR Pregnancy ended early OR Evidence of preterm delivery in the open narrative

Preterm delivery with respiratory distress syndrome

Pregnancy duration less than 9 months or Pregnancy ended early AND Fast breathing AND No fever AND No cold to touch

Sepsis (part of ‘Serious Infection’)

Fever or Cold to touch AND No diagnosis of pneumonia or meningitis

Neonatal jaundice

Yellow skin or eyes

Haemorrhagic disease of the newborn

Bleeding from anywhere

Sudden unexplained infant death

No documented illness signs or symptoms AND Appeared healthy and then died suddenly

Unspecified (Unknown)

Does not meet any of the above criteria AND No other specified diagnosis

Young child deaths (1-59 months old)

AIDS

Mother ever tested positive for HIV OR A health worker ever told the mother she had AIDS

If the responses to both of the above are ‘Refuse to answer’ or ‘Don’t know’, then use the below criteria: Child had swelling in the armpits OR Child had a whitish rash inside the mouth or on the tongue

AIDS with tuberculosis

Same as above for AIDS AND Cough AND The cough lasted more than 2 weeks

Diarrhoea

More frequent loose or liquid stools than usual

Dysentery

Visible blood in the loose or liquid stools

Haemorrhagic fever

Bleeding from anywhere OR Skin turned black

Malaria

Fever

Malnutrition (severe)

Limbs became very thin OR Swollen legs or feet OR Protruding belly

Measles

Fever AND Rash

Meningitis

Stiff neck OR Bulging fontanelle OR Generalised convulsions or fits

Pertussis

Severe cough

Pneumonia

Difficult breathing OR Fast breathing

Sepsis

Fever AND No diagnosis of pneumonia or meningitis

Note: This report uses the term ‘other infection’ to match the Expert Algorithm terminology rather than the physician minimum criteria term ‘sepsis’. The Expert Algorithm also originally had a diagnosis called ‘sepsis’ but its name was changed prior to the 2014 study to ‘other infection’ to match CHERG terminology

Tuberculosis

Cough AND The cough lasted more than two weeks

Injury (Venomous, Drowning, Fall, Fire, Poisoning, Road traffic, Violent, Unspecified)

Suffered an injury or accident

Other childhood infectious disease (not specified above)

Fever AND Had an infectious diagnosis not specified above

Malignant neoplasm

Medical records information or death certificate diagnosis

Haemorrhagic disease of the newborn (delayed)

Bleeding from anywhere

Sudden unexplained infant death

No documented illness signs or symptoms AND Illness duration = 0 days

Unspecified (Unknown)

Does not meet any of the above criteria AND No other specified diagnosis
